# Examining the temporal behavior of the hydrocarbonaceous overlayer on an iron based Fischer–Tropsch catalyst†

**DOI:** 10.1039/c8ra09731c

**Published:** 2019-01-18

**Authors:** Robbie Warringham, Alisha L. Davidson, Paul B. Webb, Robert P. Tooze, Russel A. Ewings, Stewart F. Parker, David Lennon

**Affiliations:** School of Chemistry, Joseph Black Building, University of Glasgow Glasgow G12 8QQ UK David.Lennon@glasgow.ac.uk +44-141-330-4372; School of Chemistry, University of St Andrews St Andrews KY16 9ST UK; Group Technology, Research and Technology, Sasol Ltd UK; ISIS Facility, STFC Rutherford Appleton Laboratory Chilton Didcot Oxon OX11 0QX UK

## Abstract

In order to examine fundamental processes connected with the use of an unpromoted iron based Fischer–Tropsch synthesis (FTS) catalyst, model studies examining the temporal formation of hydrocarbonaceous species that form over the catalyst are undertaken using a combination of temperature-programmed oxidation, powder X-ray diffraction, Raman scattering, transmission electron microscopy and inelastic neutron scattering (INS). Catalyst samples were exposed to ambient pressure CO hydrogenation at 623 K for defined periods of time-on-stream (3, 6, 12 and 24 h) prior to analysis. INS reveals a progressive retention of hydrogenous species that is associated with the evolution of a hydrocarbonaceous overlayer, as evidenced by the presence of sp^2^ and sp^3^ hybridized C–H vibrational modes. Correlations between the formation of aliphatic and olefinic/aromatic moieties with post-reaction characterization leads to the proposal of a number of chemical transformations that, collectively, define the conditioning phase of the catalyst under the specified set of reaction conditions. A comparison between the inelastic neutron scattering spectra of the 24 h sample with that of an iron catalyst extracted from a commercial grade Fischer–Tropsch reactor validates the relevance of the experimental approach adopted.

## Abstract 2

Inelastic neutron scattering is used to examine an un-promoted iron based Fischer–Tropsch synthesis catalyst exposed to ambient pressure CO hydrogenation at 623 K for defined periods of time-on-stream (3, 6, 12 and 24 h).

## Graphical abstract

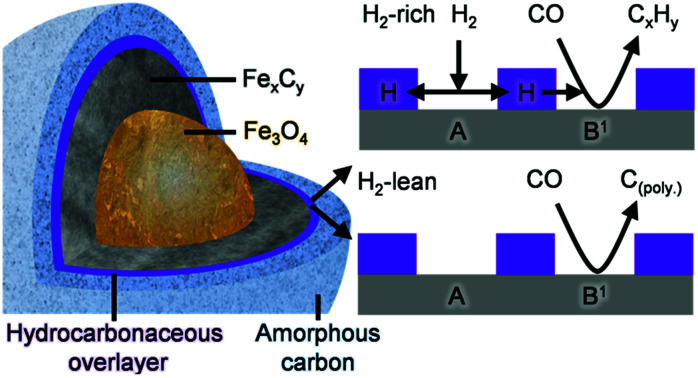

## 1. Introduction

Fischer–Tropsch synthesis (FTS) is a non-selective heterogeneously catalyzed industrial reaction that can produce a variety of hydrocarbon products from the reaction of CO and H_2_ (syngas) obtained from coal, natural gas or biomass sources.^1–3^ These hydrocarbon products may be further processed to produce sulfur-free diesel and high value chemicals utilized by the chemical manufacturing industry.^4^ If the syngas mixture is sourced from oil-free resources such as coal, FTS is considered an alternative route towards the production of fuel, thereby alleviating the dependency of crude oil derived hydrocarbons.^5,6^ Several variants of the process operate around the world, with catalysts predominantly based on Fe and Co containing materials.^3^ The high hydrogen content of natural gas is suited to Co catalysts, whereas hydrogen deficient syngas from biomass and coal is suited to Fe catalysts due to its high activity for water gas shift (WGS) chemistry.^7^

Despite its widespread industrial usage, the mechanism by which Fe-based catalysts operate is still debated in the literature. This is in part due to the complexity of the catalyst composition under reaction conditions where a mixture of iron oxides, iron carbides and carbonaceous material can be identified.^2,8–10^ Further, the reaction conditions necessary to induce FTS activity (high pressure/temperature) and the formation of high molecular weight products means *in situ* analysis of the active catalyst system is challenging. Xu and Bartholomew used high pressure *in situ* Mössbauer absorption spectroscopy to study the phase transformation of an Fe-based catalyst under realistic FTS conditions (10 atm, 538 K).^11^ Other *in situ* studies utilizing X-ray absorption spectroscopy (XAS), X-ray diffraction (XRD), and scanning transmission X-ray microscopy (STXM) have also been reported.^12–14^ An alternative approach to *in situ* analysis is to investigate the Fe catalyst undergoing ambient pressure hydrogenation of CO at elevated temperatures.^15^ Although no polymerization occurs to form the typical distribution of high molecular weight hydrocarbon products, the surface chemistry involved in CO/H_2_ dissociation and C–C/C–H bond formation is informative and relevant.

Previous studies from this group have utilized this test reaction to study how hydrogen is partitioned within the catalyst matrix of reacted Fe FTS catalysts through the application of inelastic neutron scattering (INS).^16–19^ From measuring industrially reacted and laboratory prepared Fe FTS catalysts, the formation of hydrocarbonaceous deposits during the large scale FTS operation and the representative laboratory reaction was established. A further study found that H_2_ pre-treatment before the CO hydrogenation reaction increased the level of hydrocarbonaceous species present on the sample, particularly sp^2^ hybridized carbon moieties.^18^ These investigations led to the proposal that the polyaromatic overlayer found on these reacted Fe catalysts could have a role in the mediation and transfer of reactive species to catalytic active sites.

For the present study, the temporal dependence of the hydrocarbonaceous overlayer formed on a Fe FTS catalyst during ambient pressure CO hydrogenation (H_2_ : CO = 2 : 1, 623 K) is analyzed post-reaction using a combination of characterization techniques: temperature-programmed oxidation (TPO), powder X-ray diffraction (XRD), Raman spectroscopy, transmission electron microscopy (TEM) and INS. For the first time, the INS spectra reveal a progressive retention of hydrogenous species as a function of time-on-stream that is associated with the evolution of the hydrocarbonaceous overlayer. Correlations between the formation of aliphatic and olfenic/aromatic moieties with post-reaction TPO profiles, plus other analytical probes, leads to the proposal of a number of chemical transformations that collectively define the conditioning phase of the catalyst under the specified set of reaction conditions. A comparison between the INS spectra of a 24 h reacted sample with an Fe FTS catalyst extracted from a commercial grade FTS reactor indicates the relevance of the experimental approach adopted. A qualitative model is proposed to account for the experimental observations.

## 2. Experimental

### 2.1. Catalyst preparation and characterization

The iron oxide catalyst sample used for this investigation was prepared using the co-precipitation of iron nitrate (Sigma Aldrich, 99.99%) and sodium carbonate (Sigma Aldrich, 99.99%). The preparative procedure utilizes a batch reactor apparatus for reproducible sample synthesis and is described elsewhere.^19^ The procedure produces hematite (α-Fe_2_O_3_) with a surface area of 70.8 m^2^ g^−1^ and an absence of promoters/modifiers. All samples were ground and sieved to a particle size range of 250–500 μm.

### 2.2. Micro-reactor measurements

Initial reaction testing was performed using a catalyst test line composed of 1/8 in. diameter stainless steel Swagelok tubing; a description of which can be found elsewhere.^20,21^ Approximately 40 mg of catalyst was loaded into a 1/4 in. quartz tube reactor with the reactor plugged with quartz wool. The reactor is housed within a tube furnace (Carbolite MTF 10/15/30) equipped with PID control. A thermocouple is positioned within the catalyst bed to ensure accurate temperature reading during measurements. For CO hydrogenation reactions ambient pressure gas flows of CO (3.35 sccm, CK Gas, 99.8%), H_2_ (6.75 sccm, BOC Ltd, 99.8%) and He (21.25 sccm, BOC Ltd, 99.9%) were established over the bypass before introduction to the catalyst reactor (total weight hourly space velocity (WHSV) of 60.8 h^−1^). All gas flows were monitored using an in-line quadrupole mass spectrometer (Hiden Analytical, HPR-20) attached to the reactor exit line *via* a differentially-pumped, heated quartz capillary. Under a continuous feed of reactant gases, the sample was subjected to a temperature ramp of 5 K min^−1^ to 623 K and held for a pre-determined length of time (*x*), after which the reactant flows were stopped and the sample was cooled to room temperature under the helium carrier gas. The micro-reactor samples are referred to as MR-*x*, where *x* indicates the time on stream in hours. For *ex situ* characterization, reacted samples were subjected to a passivation procedure involving the introduction of small amounts of O_2_ to the reactor, gradually increasing to atmospheric levels (*i.e.* 20% O_2_ in the gas feed).^22^ As mentioned elsewhere,^17^ electing to operate syngas reactions at ambient pressure permits access to Fe/CO/H_2_ surface chemistry whilst ensuring the C–C propagation stage is inaccessible, as the latter would otherwise lead to products that would dominate the INS spectrum. Thus, the approach adopted here enables the investigation of surface species to take place without such species being ‘swamped’ by a high molecular weight product slate.

### 2.3. Inelastic neutron scattering measurements

For INS measurements, approximately 10 g of catalyst was loaded into an Inconel reactor cell and attached to a custom built sample preparation rig.^23^ For ambient pressure CO hydrogenation measurements, the iron oxide catalyst was heated to 623 K at 5 K min^−1^ under a flow of CO (75 sccm, CK gas, 99.9%) and H_2_ (150 sccm, CK gas, 99.9%) in a carrier gas (He, 600 sccm, CK gas, 99.9%, total WHSV of 1.47 h^−1^) and held at temperature for a pre-determined length of time. The gas products were analyzed by an in-line mass spectrometer (Hiden Analytical, HPR20 QMS Sampling System). Note that the MS instrument utilized for these scaled-up reaction measurements at the Central Facility is uncalibrated at the time of measurement, therefore the gas traces are a qualitative representation of the reaction profiles. Once the specific reaction had finished, the reactant gases were stopped and the sample allowed to cool to room temperature under the carrier gas. The samples prepared in the large scale reactor are referred to as LR-*x*, where *x* indicates the time on stream in hours. The reactor cell was isolated and placed in the argon-filled glove box (MBraun UniLab MB-20-G, [H_2_O] <1 ppm, [O_2_] <2 ppm) before being loaded into an aluminum sample holder that is then sealed *via* an indium wire gasket.^24^ All INS measurements were performed using the MAPS direct geometry spectrometer.^25^ Spectra were recorded at 20 K at an incident neutron energy of 600 meV and 250 meV using the A-chopper package. Quantification of the *ν*(C–H) feature obtained by INS was achieved following a calibration protocol described elsewhere.^26^ Briefly, samples containing known masses of a polystyrene calibrant (Sigma Aldrich, >99.0%, typical molecular weight = 350000) were analyzed using the MAPS spectrometer at an incident energy of 600 meV. The resulting spectra were baseline corrected and fitted with Gaussian functions using the Origin graphical software package (MicroCal Origin, Version 8.0, Fig. S1†). The integrated response was related to hydrogen content of the calibrant material; the calibration plots are presented in the ESI (Fig. S1†).

### 2.4. Post-reaction analysis

TPO of the micro-reactor samples was performed post-reaction *in situ* whilst the large scale reactor was performed *ex situ*. Oxygen (5% in He, 70 sccm, BOC Ltd, 99.5%) was introduced to the sample (*ca.* 40 mg) and the reactor heated to 1173 K at 5 K min^−1^ using the mass spectrometer to monitor the eluting gases. Quantification of the CO_2_ peak area was achieved by measuring the CO_2_ response from the *in situ* TPO of known masses of graphite (Sigma Aldrich, 99.9%).^27^ Powder XRD was performed using a Siemens D5000 diffractometer, with a Cu Kα radiation in Bragg–Brentano geometry in the 2*θ* range 5–85° (step size 0.02° s^−1^). For *in situ* XRD studies *ca.* 200 mg of ground sample was placed in an Anton Paar XRK-900 reaction chamber with a K-type thermocouple was housed in the reaction chamber. Temperature control was maintained by an Anton Paar TCU 750 temperature control unit equipped with a PID control (Eurotherm 2604). A H_2_ : CO mixture (2 : 1, 10 sccm, CK Gases, 99.5%) in carrier gas (Ar, 20 sccm, BOC Ltd, 99.9%) were introduced *via* 1/4 in. Swagelok tube gas lines, with a thermocouple positioned within the catalyst bed to ensure accurate temperature reading during measurements. The sample was heated to 623 K at 5 K min^−1^ and maintained at 623 K for 24 h. Diffractograms were recorded every hour. Reflections were assigned based on the following reference diffraction patterns; α-Fe_2_O_3_, JCPDS #13-534; Fe_3_O_4_, JCPDS #19-629; Fe_5_C_2_, JCPDS #36-1248; Fe_3_C, JCPDS #32-0772. *Ex situ* Raman scattering was performed using a Horiba Jobin Yvon LabRam HR confocal Raman microscope and a 532 nm laser source at <20 mW power. Measurements were taken for approximately 5 min. TEM was performed using a Tecnai T20 microscope equipped with a post-column Gatan Imaging Filter and operated with an accelerating voltage of 200 keV. Elemental maps were produced using energy filtered TEM (EFTEM), adopting the three window technique to distinguish the O–K, C–K and Fe-L2.3 EELS edges from a power law background. Samples were ground and suspended in MeOH (Sigma Aldrich, 99.8%) before deposition on a holey carbon grid and dried for insertion in to the microscope chamber.

## 3. Results and discussion

### 3.1. Micro-reactor characterization

**Fig. 1:**
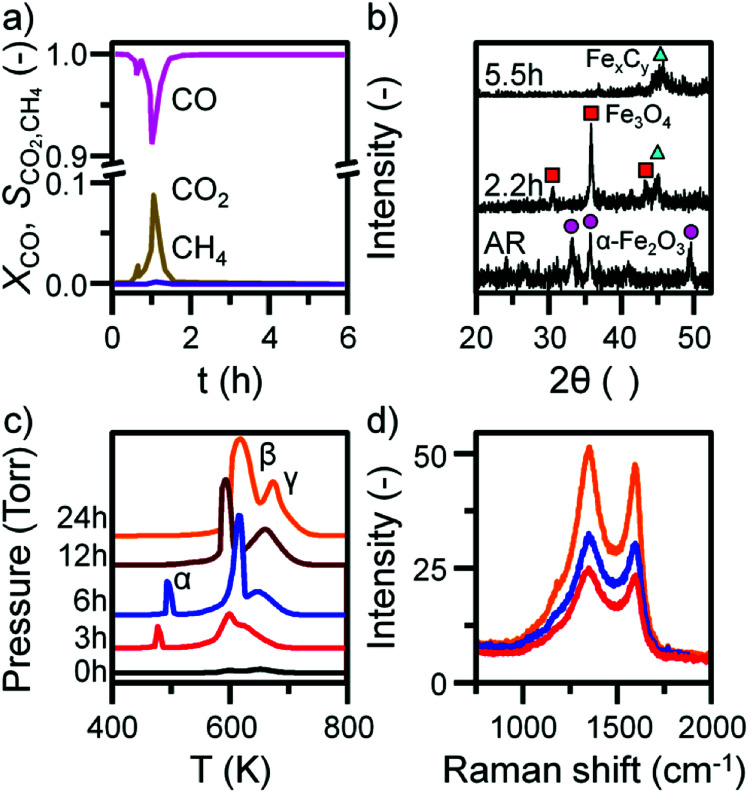
(a) The conversion of CO and selectivity to CO_2_ and CH_4_ over the α-Fe_2_O_3_ sample during CO hydrogenation at 623 K in the micro-reactor. (b) *In situ* XRD with identified reflections attributed to α-Fe_2_O_3_ (circle), Fe_3_O_4_ (square), and iron carbides (triangle). (c) *In situ* TPO MS profiles and (d) selected *ex situ* Raman spectra of the α-Fe_2_O_3_ sample after CO hydrogenation at 623 K in the micro-reactor. TPO profiles are stacked to facilitate comparison.

Previous characterization efforts have established the phase of the iron oxide catalyst sample, investigated for this study, to be α-Fe_2_O_3_.^19^ Those studies also discussed the reaction chemistry during the representative FTS reaction (ambient pressure CO hydrogenation at 623 K for 6 h).^19^ The focus for this particular investigation is on the temporal behavior of the hydrocarbon retention process during CO hydrogenation. The reaction was repeated for several samples whilst varying the time-on-stream (0, 3, 6, 12, and 24 h). Fig. S2† presents the reaction profile obtained after 24 h. As for the previously reported 6 h reaction,^19^ three stages are identified: the reduction of the α-Fe_2_O_3_ to Fe_3_O_4_ by CO to produce CO_2_ (Stage I), the simultaneous production of CO_2_, H_2_O, and CH_4_ with a corresponding consumption of CO and H_2_ at 623 K (Stage II), and a decrease in product yield towards steady-state operation (Stage III). A degree of WGS activity could additionally be contributing to the observed reaction profile. CO conversion approximates to <1% and is minimal during the course of the reaction measurement, corresponding to an iron time yield of 1.6 × 10^−5^ mol CO g_Fe_^−1^ s^−1^ (Fig. 1a). This is a comparable rate to values reported for similar reaction conditions.^28^ The catalyst retains this low activity throughout the course of the 24 h reaction without deactivation. *In situ* XRD identifies the morphological transformations during this period, with the reduction of α-Fe_2_O_3_ to Fe_3_O_4_ leading to the formation of iron carbides (Fig. 1b). It is noted that the gas exchange characteristics of the *in situ* XRD cell are different to the micro-reactor set-up (not least, a larger sample mass in the *in situ* XRD reaction cell). This is thought to lead to a prolongation of the reduction period, *i.e.* Stage I (Fig. S2†), with the consequence that the presence of iron oxide phases persists for longer in the XRD cell than occurs in the micro-reactor. From 6.6 h onwards (not shown) there was a substantial decrease in signal-to-noise attributed to carbon build-up.

**Fig. 2:**
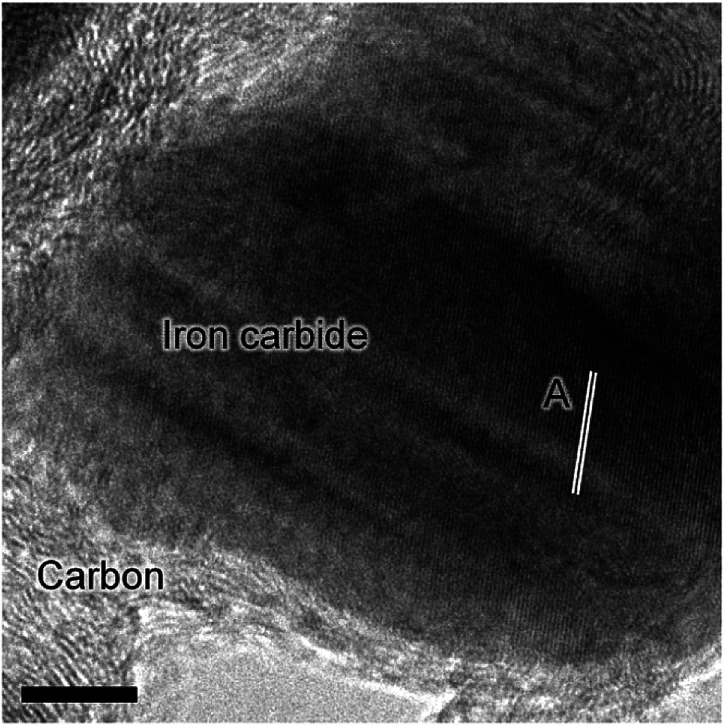
*Ex situ* HRTEM micrograph of the post-reaction (24 h) α-Fe_2_O_3_ sample.

*In situ* TPO has been demonstrated to be a useful tool in discriminating and quantifying transient carbon species formed under CO hydrogenation conditions at 623 K.^19^ From our previous study, three peaks were observed after 6 h on-stream and were assigned to reactive adsorbed carbon (α), amorphous-like carbon (β) and iron carbides (γ) (Fig. 1c).^19^ Here we observe the formation of these peaks occurring between 0 to 3 h at 623 K (Fig. 1c), concurrent with the reduction of α-Fe_2_O_3_ to Fe_3_O_4_ and beginning of iron carbide formation. Therefore, we propose the origins of these peaks are related with carbon deposition *via* a combination of CO dissociation/disproportionation and carbide formation, indicative of the 'evolutionary' nature of iron oxide FTS catalysis as discussed by Schulz.^29^Table 1 gathers the quantified peak areas for the α, β and γ peaks. After 6 h on-stream, the α peak is lost, whilst the β and γ peaks remain. The β peak, attributed to amorphous carbon, continues to increase in mass during the course of the reaction. The γ peak, ascribed to iron carbide species, levels after about 12 h on-stream. The increase in carbon retention is also observable by Raman spectroscopy (Fig. 1d) and, combined with the CO conversion, *in situ* XRD and TPO data, indicates that the carbon speciation is still in dynamic flux after 24 h reaction. Visualization of the 24 h reacted sample by *ex situ* TEM identifies a core shell structure containing a thick outer layer of approximately 5 nm (Fig. 2), consistent with other microscopy studies of reacted iron FTS catalysts.^17,19,30–32^ The lattice fringes observable within the central core (site A) were found to be 0.190 nm, consistent with iron carbide (*e.g.* Hägg carbide, *d*(600) = 0.191 nm),^18^ whilst the fringes surrounding the core suggest a graphitic character to the encapsulating carbon. Energy filtered micrographs confirm the presence of iron and carbon throughout the cores, without significant oxygen content. Low levels of oxygen are observed, however, within a minority of small dense regions that also contain iron but are smaller than the dominant nanoparticles (Fig. S3†).

**Table 1:** A comparison of the quantified peak area and temperature max from the TPO studies involving samples from the micro-reactor and large scale reactor samples

Sample	α peak		β peak		γ peak	
	C content^c^	*T*_max_ d	C content	*T*_max_	C content	*T*_max_
MR-0^a^	—		1.04	600	2.07	653
MR-3	3.32	477	13.83	600	5.89	628
MR-6	4.14	494	16.81	611	8.04	649
MR-12	—		18.09	592	18.51	660
MR-24	—		33.43	618	24.04	674
LR-3^b^	2.65	465	16.80	585	2.50	619
LR-6	2.69	482	18.42	595	7.30	629
LR-12	10.13	487	23.41	597	5.95	630
LR-24	15.98	500	26.58	606	11.66	639

a MR – micro-reactor.

b LR – large scale reactor.

c Carbon content in mmoles_C_.

d *T*_max_ in K.

These types of structure were also reported in our previous studies and could either represent unreduced iron oxide or metallic iron which has re-oxidized under the passivation conditions exposed to the catalyst before the *ex situ* analysis.^17,19^

### 3.2. Large scale reactor characterization

**Fig. 3:**
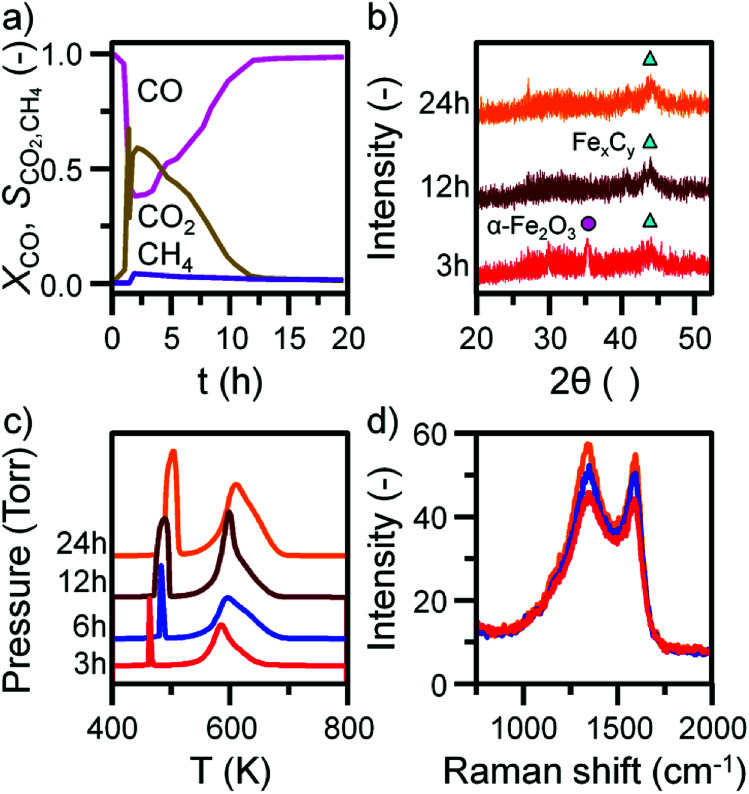
(a) The conversion of CO and selectivity to CO_2_ and CH_4_ over the α-Fe_2_O_3_ sample during CO hydrogenation at 623 K in the large scale reactor. (b) *Ex situ* XRD with identified reflections attributed to α-Fe_2_O_3_ (circle), and iron carbides (triangle). (c) *Ex situ* TPO MS profiles and (d) selected *ex situ* Raman spectra of the α-Fe_2_O_3_ sample after CO hydrogenation at 623 K in the large scale reactor. TPO profiles are stacked to facilitate comparison.

Before INS analysis, the α-Fe_2_O_3_ sample was exposed to scaled-up CO hydrogenation conditions using a custom built apparatus for the preparation of large catalyst samples (*ca.* 10 g) suitable for INS analysis.^23^ The MS reaction profile for the 24 h reacted sample is presented in Fig. S4.† As with the micro-reacted sample (Fig. S2†), the large scale reaction profile can be defined by several stages: the simultaneous production of CO_2_, H_2_O and CH_4_ once at the reaction temperature, followed by pseudo-steady state operation after approximately 12 h on-stream. It should be noted no wax products were formed during the course of this measurement, confirming that the selected operational regime does not induce the formation of long chain hydrocarbons usually associated with the FTS process that is favored at elevated pressures. The primary route of formation of CO_2_ in the micro-reactor set-up was the reduction of α-Fe_2_O_3_ to Fe_3_O_4_ by CO. From the selectivity: conversion profile in Fig. 3a the sharp decrease in CO_2_ signal coincides with the CO signal approaching steady-state. This would indicate the α-Fe_2_O_3_ has been reduced to Fe_3_O_4_, decreasing the routes of CO_2_ production. Reflecting the larger sample mass utilized for the large scale reaction (10 g *versus* 40 mg in the micro-reactor), longer run times are required to fully reduce the α-Fe_2_O_3_ starting material when compared with the micro-reactor arrangement. *Ex situ* XRD indicates the complete reduction of α-Fe_2_O_3_ from 12 h (Fig. 3b). Further, there is an increase in iron carbide reflections between 40 and 50° up to 24 h. The increase is less than observed for the *in situ* measurement where, after approximately 5 h on-stream, the diffractogram was dominated by iron carbide features (Fig. 1b). This observation is consistent with the inferior solid/gas exchange dynamics of the INS reactor.

In contrast to the micro-reactor TPO profiles (Fig. 1c), the TPO profiles obtained for the large scale reactor samples are similar in profile shape; the α peak is sharp and defined, whilst the β peak is broad, containing a high temperature shoulder (γ peak, Fig. 3c). The quantified peak areas are collected in Table 1. As with the micro-reactor studies, carbon retention increases during the course of the reaction, particularly the reactive adsorbed carbon associated with the α peak.

This species is only present for the first 6 h of reaction in the micro-reactor (Fig. 1c), suggesting the large scale operation enables this carbon species to persist for longer periods of time-on-stream consistent with the inferior gas/solid exchange dynamics. *Ex situ* Raman spectra are defined by the ‘D’ and ‘G’ bands indicative of disordered and ordered carbonaceous deposits (Fig. 3d).^33–35^ The small increase in intensity from 3 to 24 h is consistent with the increase in carbon retention observed by TPO (Fig. 3c, Table 1).

### 3.3. Inelastic neutron scattering studies

**Fig. 4:**
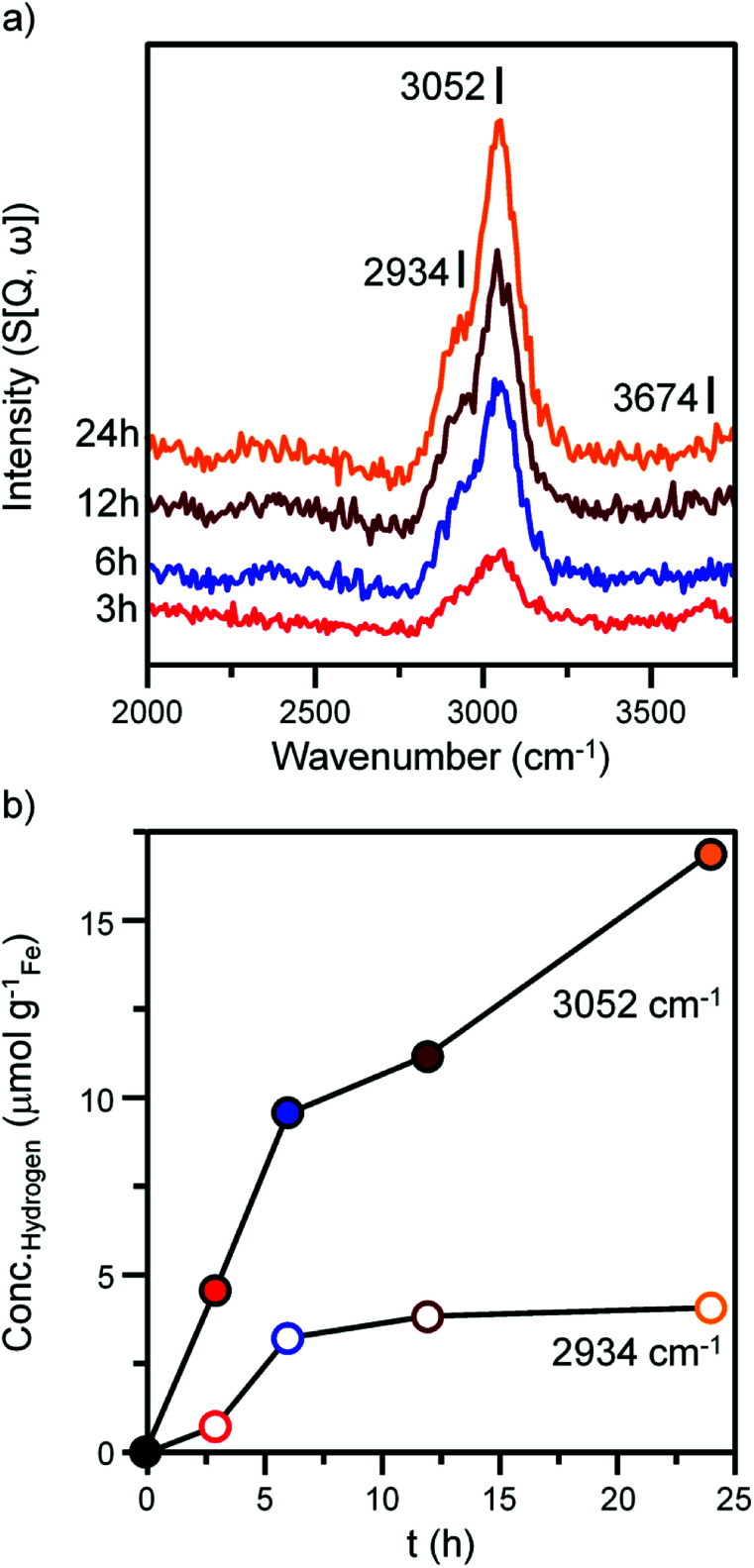
(a) INS spectra (recorded at 600 meV) of the α-Fe_2_O_3_ after CO hydrogenation at 623 K in the large scale reactor. Spectra are stacked to facilitate comparison. (b) The quantified hydrogen content of the 2934 cm^−1^ (hollow) and 3052 cm^−1^ (solid) features identified in part (a).

**Fig. 5:**
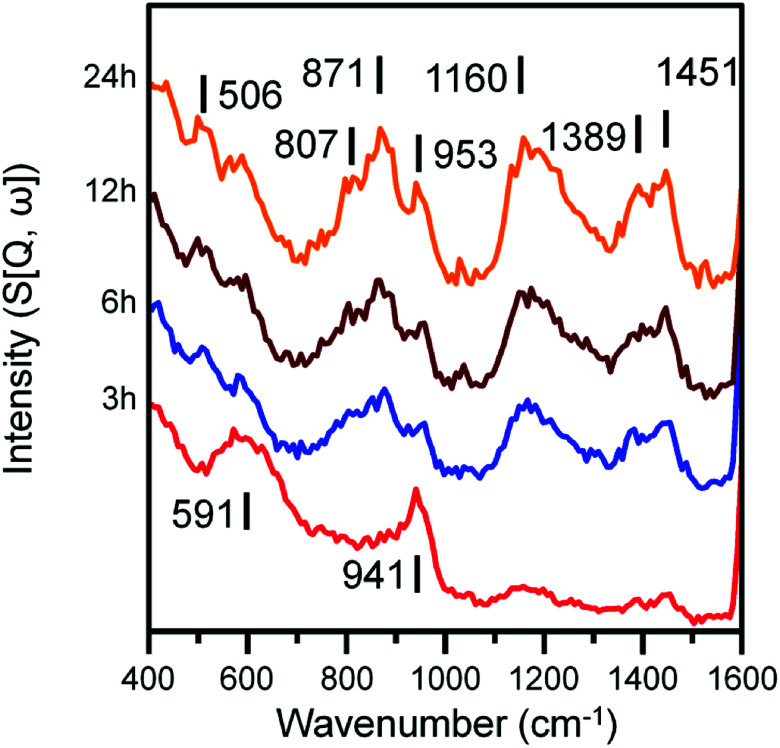
INS spectra (recorded at 250 meV) of the α-Fe_2_O_3_ sample after CO hydrogenation at 623 K in the large scale reactor. Spectra are stacked to facilitate comparison.

Fig. 4 and 5 presents the INS spectra of the reacted samples recorded at an incident energy of 600 and 250 meV respectively. At 600 meV, the spectra are defined by the presence of a *ν*(C–H) feature consisting of a main peak at 3052 cm^−1^ with a noticeable shoulder at *ca.* 2934 cm^−1^ (Fig. 4a). These bands are assigned to the *ν*(C–H) modes of sp^2^ and sp^3^ hybridized carbon.^18,19^ There is a small but noticeable peak at 3674 cm^−1^ for the 3 h reacted sample attributed to the *ν*(O–H) mode of terminal hydroxyl groups. With time-on-stream the intensity of the *ν*(C–H) features increase, whilst the *ν*(O–H) mode is not present from 6 h onwards indicating a progressive deposition of hydrocarbonaceous material and the removal of the small population of surface hydroxyls.

An advantage of INS is the spectral intensity for a specific mode is directly proportional to the hydrogen concentration.^36^ Integration and quantification of the *ν*(C–H) features *via* described calibration procedures then enables mode specific hydrogen retention as a function of time-on-stream to be established (Fig. S4b and S5†).^26^ The aliphatic sp^3^ hybridized C–H mode increases during the initial stages of reaction before saturating after *ca.* 8 h. In contrast, olefinic/aromatic sp^2^ hybridized C–H species continually increase during the reaction. Correlating the quantified INS trends with the TPO peak profiles (Fig. 3c, Table 1) suggest the sp^2^ hybridized C–H entities are related to the steadily increasing β peak, *i.e.* amorphous carbon. However, the sp^3^ hybridized *ν*(C–H) signal does not correlate with any of the α, β or γ peaks present in Fig. 3c. It is possible that desorption or partial oxidation events could be affecting the TPO profiles.

Spectra recorded at an incident energy of 250 meV are presented in Fig. 5. Several weak features are present after 3 h on-stream (1451, 1389 and 1160 cm^−1^) that are assigned to aromatic *δ*(C–H) modes.^16–19^ The 1451 cm^−1^ feature is assigned to semi-circle ring deformation modes that are possibly linked with a *δ*(C–H) mode associated with perimeter carbons of an extended polycyclic aromatic network.^37^ The 1389 cm^−1^ band is assigned to an in-plane ring deformation of a naphthalene-type molecule and the 1160 cm^−1^ band is assigned to a CC–H in-plane deformation mode of a polyaromatic hydrocarbon. The relatively weak intensity of these features suggests the presence of aromatic species at this stage of the reaction is relatively low. The remainder of peaks at 3 h can be assigned to the A_1g_ Fe–O phonon mode of Fe_3_O_4_ (591 cm^−1^) and a combination of alkenic *δ*(C–H) and a possible magnetic interaction associated with Fe_3_O_4_ (941 cm^−1^).^19,38^ With time-on-stream, the intensity of the sp^2^ hybridized carbon features between 1000 to 1500 cm^−1^ increase. The signals observed at 871 and 801 cm^−1^ for the 6, 12 and 24 h reacted sample spectra can be assigned to the out-of-plane C–H deformations of either an olefinic or aromatic group.^39^ The band at 506 cm^−1^ is assigned to a C–C torsion mode of edge carbon atoms contained within a polycyclic aromatic network.^37^

### 3.4. Proposed scheme

**Fig. 6:**
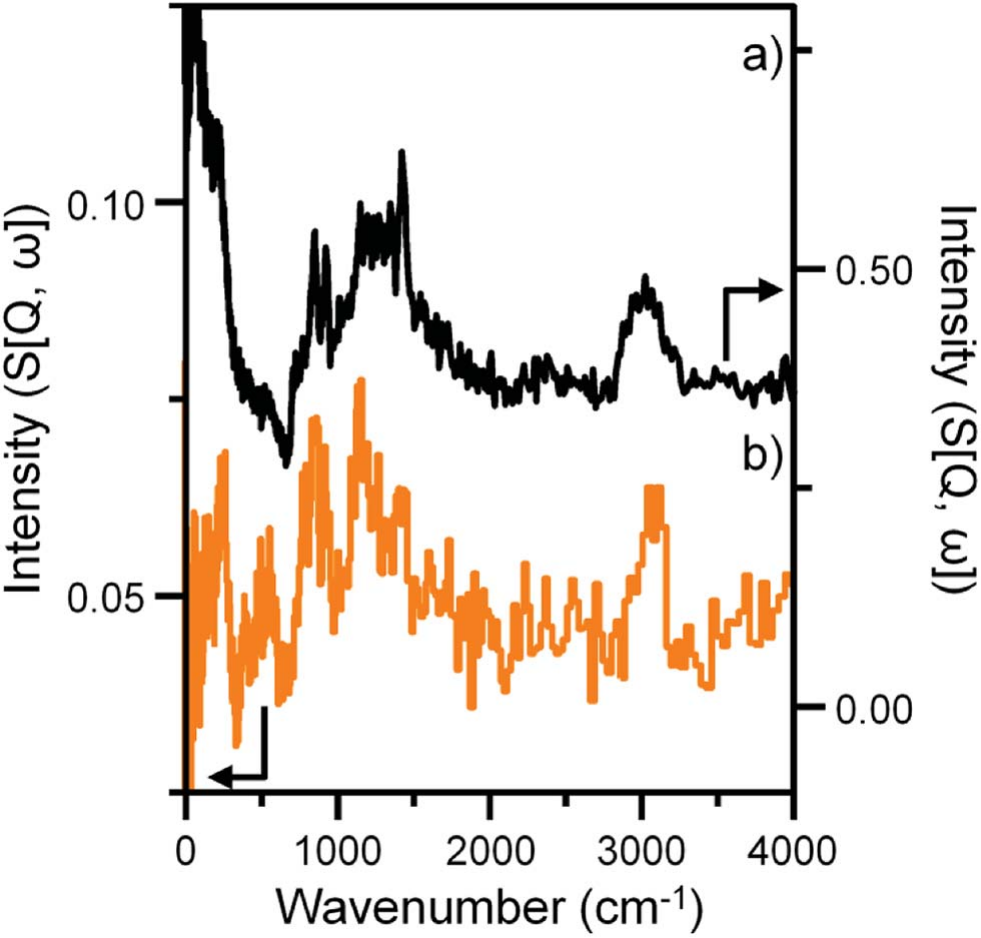
INS spectral comparison of (a) a reacted commercial grade Fe FTS catalyst after solvent extraction^16^ and (b) the α-Fe_2_O_3_ sample after CO hydrogenation at 623 K for 24 h using the large scale reactor set-up. Both spectra were obtained with the TOSCA spectrometer.

**Fig. 7:**
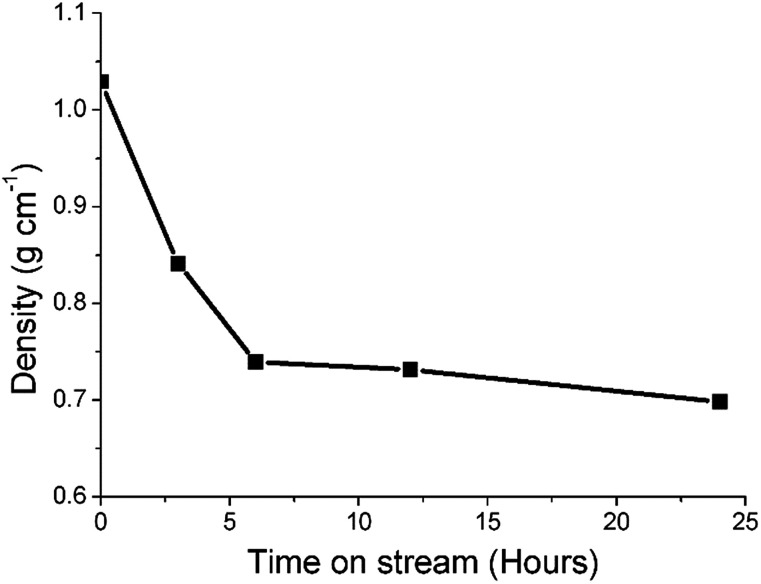
A plot of density of the catalyst extracted from the large scale INS reactor as a function of time-on-stream (CO hydrogenation at 623 K).

Previous studies from this group have investigated an industrially reacted iron based FTS catalyst by INS using the TOSCA instrument.^16^ The spectrum is indicative of hydrocarbonaceous deposits consisting of a polyaromatic overlayer that retains some aliphatic character.^16^ As the MAPS spectrometer is characteristically different from the TOSCA spectrometer (direct *versus* indirect geometry instruments),^25^ the 24 h reacted sample was also analyzed using the TOSCA spectrometer for direct comparison with the industrially reacted iron sample (Fig. 6). The spectra exhibit comparable characteristics, particularly in the deformation region between 800 to 1500 cm^−1^. These similarities indicate hydrocarbonaceous components present on each set of samples are composed of similar material, namely a polyaromatic over-layer possessing some aliphatic functionality. Moreover, Fig. 7 shows how the density of the catalyst extracted from the large scale INS reactor is modified as a function of time-on-stream. Catalyst density is seen to decrease sharply over the period 0–6 h before levelling off at longer reaction times. van der Loosdrecht and co-workers report iron based FTS catalysts to exhibit a decrease in density during reaction; a matter that causes problems in the process environment.^3^ To a first approximation, the trend evident in Fig. 7 is a further indication on the relevance of the selected test regime connects with changes in catalyst structure and composition as encountered in the industrial scenario.

Niemantsverdriet's respected competition model states considers the rates of FTS activity with respect to that of carbide formation.^8,40^ Here, we adopt those concepts but shift the emphasis to the not unimportant matter of hydrogen supply. Eqn (1) describes the reaction between chemisorbed carbon and hydrogen atoms to form C–H products. Under the reaction conditions utilized for this study, it is suggested that the formation of amorphous carbon (eqn (2)) is a result of a hydrogen deficiency within the reaction system, where adsorbed carbon is produced *via* the dissociative adsorption of CO. It is assumed that the carbon resides at an iron carbide site that supports dissociative adsorption of CO.^10^ Adsorbed hydrogen atoms diffuse to this site and form C–H bonds. Whilst a rich hydrogen supply will convey saturated C–H bonds, a constrained hydrogen supply will lead to unsaturated C–H bonds. Similarly, the formation of amorphous/graphitic carbon (C_(poly)_) or hydrocarbon depends on the hydrogen supply.^8,40^

1 *x*C_(ad)_ + *y*H_(ad)_ → C_*x*_H_*y*_

2 *n*C_(ad)_ → C_(poly)*n*_

**Fig. 8:**
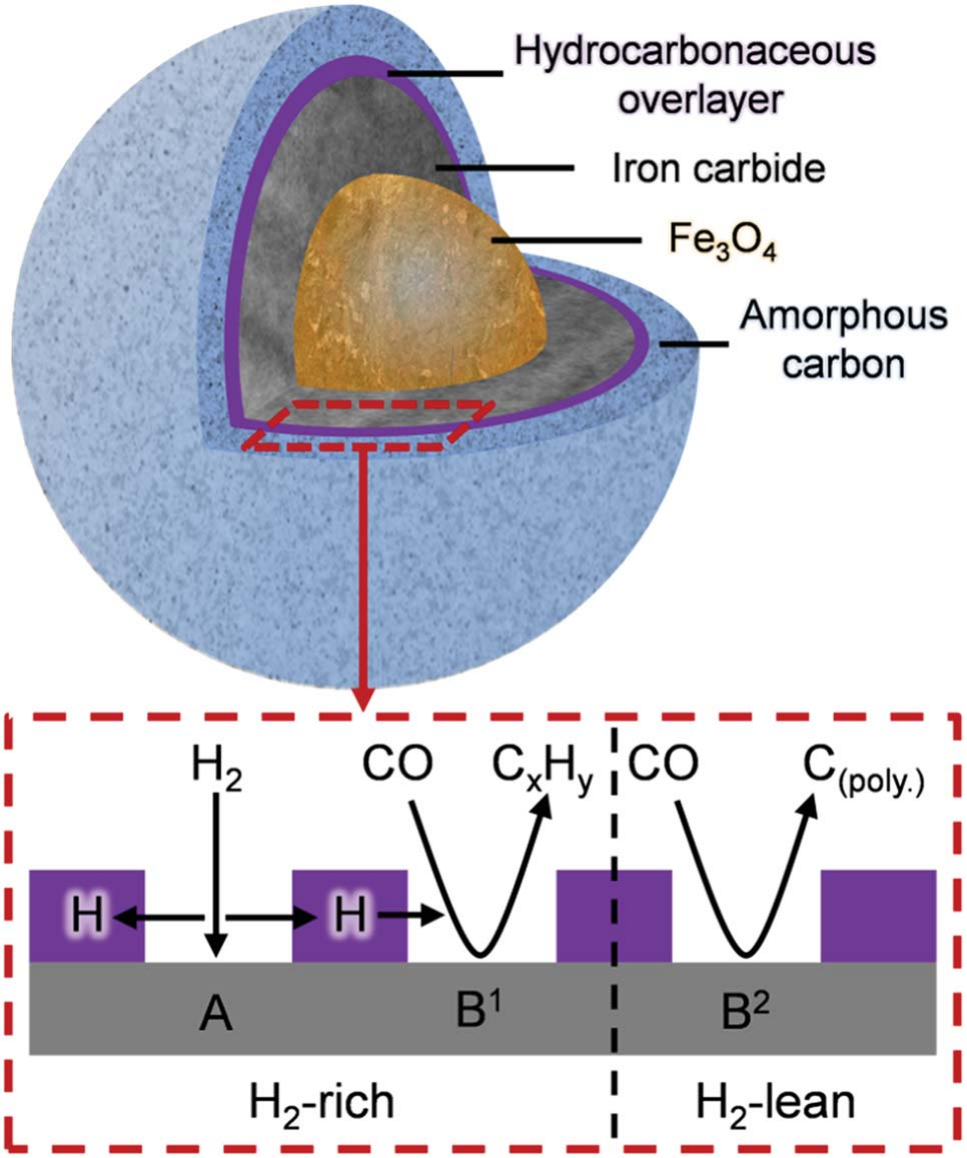
A schematic indicating the composition of the Fe FTS catalyst after *ca.* 6 h ambient pressure CO hydrogenation at 623 K. The hydrocarbonaceous overlayer (highlighted area) is suggested to mediate the transfer of hydrogen, where under H_2_-rich conditions, C_*x*_H_*y*_ prevails (site B^1^) whilst under H_2_-lean conditions, carbon polymerization occurs (site B^2^).

Fig. 8 is schematic representation that elaborates on a previously suggested model.^16^ Roles have now been included for (i) a hydrocarbonaceous overlayer and (ii) the effect of hydrogen supply. Sites for the dissociation of CO and H_2_ are identified with the hydrocarbonaceous overlayer acting as a mediator for the supply of carbon and hydrogen atoms from these sites. The following statements represent a hypothesis, illustrated in Fig. 8, which seeks to describe how the catalyst conditioning regime results in an active catalytic material capable of sustaining reagent turnover. Firstly, in the reducing environment of a syngas feed at elevated temperatures, the α-Fe_2_O_3_ pre-catalyst is reduced to Fe_3_O_4_ which then induces carbide formation. At this stage, as indicated in Fig. 4 and 5, a hydrocarbonaceous overlayer forms. It is assumed that this overlayer forms at the surface of the iron carbide.^17^ Adopting a similar line-of-thought to that considered for certain hydrogenation reactions,^41^ the coverage of the hydrocarbonaceous overlayer is partial, leaving two distinct ‘open’ sites: A and B. Site A is associated with the dissociative adsorption of hydrogen that provides a continuous source of hydrogen atoms. Site B is connected with the dissociative adsorption of carbon monoxide to produce chemisorbed carbon and oxygen atoms. Reaction of adsorbed oxygen atoms at this site with either carbon or hydrogen will result in the formation of, respectively, CO_2_ and H_2_O. Alternatively, site B could support reactions described by eqn (1) (site B^1^) and 2 (site B^2^). Under CO hydrogenation conditions (elevated temperature but low pressure), this will lead to CH_4_ formation provided the hydrogen supply is sufficient (eqn (1), site B^1^). A constrained hydrogen supply will favor the formation of amorphous/graphitic carbon (eqn (2), site B^2^). Under classical FTS conditions (elevated temperature and high pressure), eqn (1) is supplemented by a C–C propagation reaction; a rich and dynamic hydrogen supply to this site will then support formation of high molecular weight saturated products. The hydrocarbonaceous overlayer may therefore represent the precursor to both desired FT product and carbonaceous, or hard, carbon. The fate of the hydrocarbonaceous overlayer will be dependent on hydrogen supply with hard carbon formation occurring under hydrogen lean conditions.

Clearly, further work is required to evaluate the feasibility of the proposed hypothesis but, nevertheless, it can be used to rationalize established trends of Fe based catalysts applied to CO hydrogenation and FTS reactions. The novelty of this particular work is that the INS spectra show, for the first time, that the formation of a hydrocarbonaceous overlayer is an integral part of the catalyst conditioning phase of this intricate catalytic system. Moreover, the INS spectrum of the 24 h sample is comparable to a commercial grade FTS catalyst extracted from a large-scale coal-to-liquids reactor indicating that a hydrocarbonaceous overlayer is present in both samples (Fig. 6). This outcome validates the use of CO hydrogenation over a representative iron based FTS catalyst as a test reaction compatible with the acquisition of informative INS spectra.

## 4. Conclusions

CO hydrogenation has been investigated as a function of time over an un-promoted Fe based FTS catalyst and characterized using TPO, XRD, Raman, TEM and INS. The following conclusions can be drawn.

• A temporal profile is constructed from the TPO and INS studies for both the carbonaceous and hydrocarbonaceous components of the reacted catalyst samples.

• TPO profiles are assigned to reactive carbon species, amorphous carbon and iron carbide species; the development of which are dependent on the gas/solid exchange dynamics afforded by either a micro-reactor or a large scale INS reactor.

• INS analysis of the reacted catalyst samples identify the presence of sp^2^ and sp^3^ hybridized C–H species, with quantification of the *ν*(C–H) modes indicating the concentration of aliphatic C–H species to saturate after approximately 6 h time-on-stream. In contrast, the concentration of olefinic/aromatic C–H entities progressively increase as a function of time-on-stream.

• The INS spectrum for a catalyst that has experienced a 24 h reaction period yields a comparable INS spectrum to that of a commercial grade Fe based FTS catalyst that has been extracted from a large scale coal-to-liquids unit operation. This establishes the relevance of the hydrocarbonaceous overlayer concept to FTS and, moreover, indicates that the test reaction explores aspects of iron oxide/CO/H_2_ surface chemistry that has tangible connectivity to the economically relevant process chemistry.

• A schematic illustration (Fig. 8) is presented to define the range of components and probable distribution of sites connected with an active Fe based FTS catalyst.

## Conflicts of interest

There are no conflicts to declare.

## Supplementary Material

RA-009-C8RA09731C-s001
