# Supplementary material for: Examining the temporal behavior of the hydrocarbonaceous overlayer on an iron based Fischer–Tropsch catalyst
Source: RSC Adv. 2019 Jan 18;9(5):2608–17. doi: 10.1039/c8ra09731c (PMC9059844; doi:10.1039/c8ra09731c)
Supplement: RA-009-C8RA09731C-s001 [file RA-009-C8RA09731C-s001.pdf]

Electronic Supplementary Material (ESI) for RSC Advances.
This journal is © The Royal Society of Chemistry 2019

Electronic Supplementary Information

Examining the temporal behavior of the hydrocarbonaceous overlayer on an iron based Fischer-Tropsch catalyst

Robbie Warringham[1], Alisha L. Davidson[1], Paul B. Webb*[2], Robert P. Tooze[3], Russel A. Ewings[4], Stewart, F. Parker[4], David Lennon*[1]

[1]School of Chemistry, Joseph Black Building, University of Glasgow, Glasgow, G12 8QQ, UK

[2]School of Chemistry, University of St Andrews, St Andrews,KY16 9ST, UK

[3]Group Technology, Research and Technology, Sasol Ltd.

[4]ISIS Facility, STFC Rutherford Appleton Laboratory, Chilton, Didcot, Oxon OX11 0QX, UK.

1. Experimental

1.1 INS Calibration

Known weights of polystyrene (Sigma Aldrich, M_w = 350000, M_n = 170000), listed in **Table S1**, were prepared and analysed using the MAPS spectrometer at the ISIS facility, Rutherford Appleton Laboratory. An incident energy of 600 meV was used for polystyrene with spectra measured for approximately 150 µAmps. Spectra were measured using the 'A' chopper package.

Post-reaction the data was handled using OriginPro 8.0. Spectra were baseline corrected and the corresponding ν (CH) features integrated. The total number of hydrogen atoms present in each sample was calculated using the following expression;

(Mass of sample/ Mass of 1 mole) x %H per mole x N_A = Total number of H atoms

2.0 Results and Discussion

2.1 INS Calibration

Fig. S1 presents the data obtained from the polystyrene sample set measured using the 'A' chopper package with the spectra presented in **Fig. S1(A)** and the calibration plot presented in **Fig. S1(B)**. The ν (CH) for both the aliphatic and aromatic groups of polystyrene are clearly present in **Fig. S1(B)**, with the aliphatic ν (CH) at 2899 cm^{-1} and the aromatic ν (CH) at 3041 cm^{-1}. The total integrated area for these features was measured and compared to the hydrogen content of the investigated samples to produce the calibration plot in **Fig. S1(B)**. The fitted linear regression line, fixed through the origin yields an R^2 value of 0.99866.

Table S1 – A list of the sample weights used for the calibration runs using the MAPS spectrometer.

Chemical	Weight (mg)
Polystyrene	39
	131
	296
	596
	908

3.0 Figure List

Figure S1 – (A) The baseline corrected INS spectra of varying masses of polystyrene recorded using the MAPS spectrometer at an incident energy of 600 meV. The spectra were obtained using the 'A' chopper package. (B) The linear least squares fit (constrained through the origin) for the values obtained from integration of the polystyrene ν (CH) features in **Fig. S1(A)** comparing the integrated response against the number of hydrogen atoms present. The curve presents an R^2 value of 0.99866.

Figure S2 – The reaction profile for micro-reactor CO hydrogenation over the iron oxide catalyst at 623 K for 24 h. The mass traces are labelled on the right hand side in descending order. The Roman numerals indicate the different stages present within the reaction coordinate.

Figure S3 – (a) Ex situ zero energy loss TEM micrograph of the micro-reacted iron oxide catalyst after CO hydrogenation at 623 K for 24 hours with accompanying energy filtered TEM micrographs presenting the (b) iron map, (c) carbon map and (d) oxygen map. The white scale bar in the zero energy loss micrograph indicates a length of 20 nm, whilst it represents a length of 26 nm in the energy filtered micrographs.

Figure S4 – The reaction profile for scaled-up CO hydrogenation over the iron oxide catalyst at 623 K for 24 h. The mass traces are labelled on the right hand side in descending order.

Figure S5 – INS spectra of the iron oxide catalyst after CO hydrogenation at 623 K for 24 hours with Gaussian peak fittings indicating the sp^2 and sp^3 hybridised carbon contributions to the spectra.

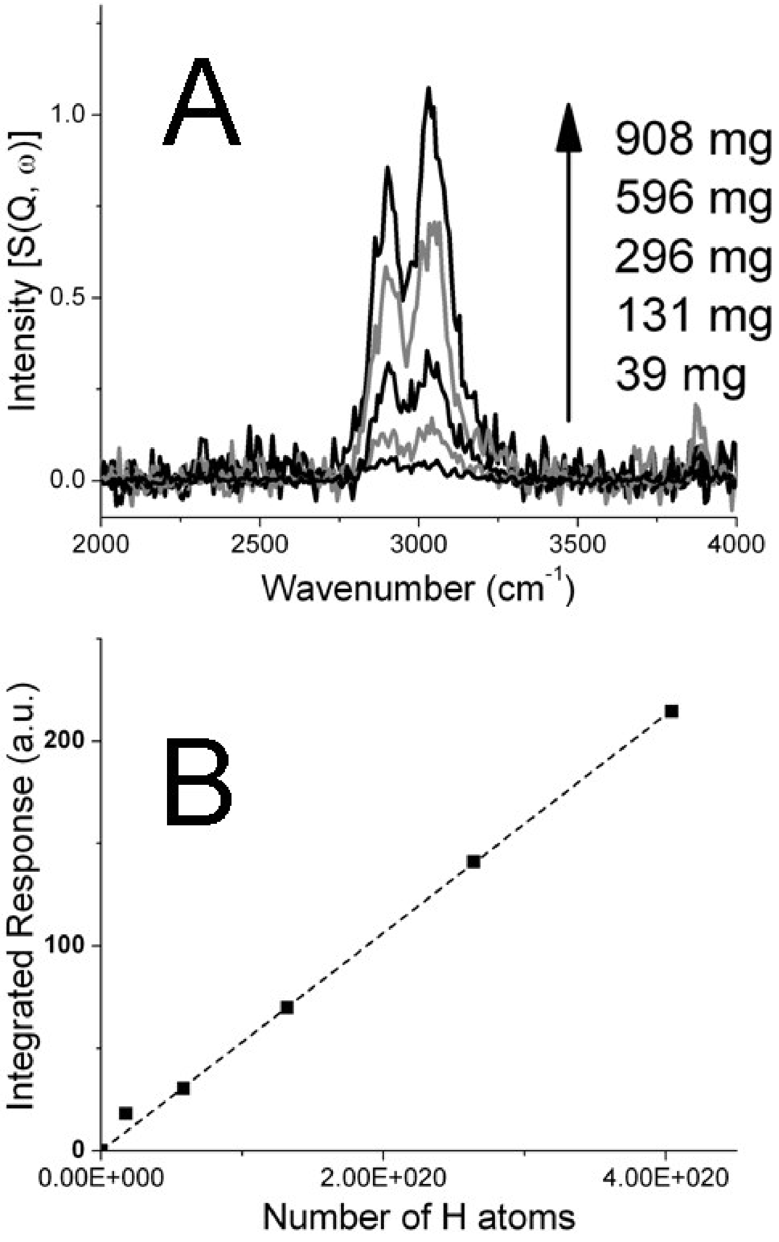

Figure S1 – (A) The baseline corrected INS spectra of varying masses of polystyrene recorded using the MAPS spectrometer at an incident energy of 4840 cm^{-1}. The spectra were obtained using the 'A' chopper package configuration. (B) The linear least squares fit (constrained through the origin) for the values obtained from integration of the polystyrene ν (CH) features in **Fig. S1A** comparing the integrated response against the number of hydrogen atoms present. The curve presents an R^2 value of 0.99866.

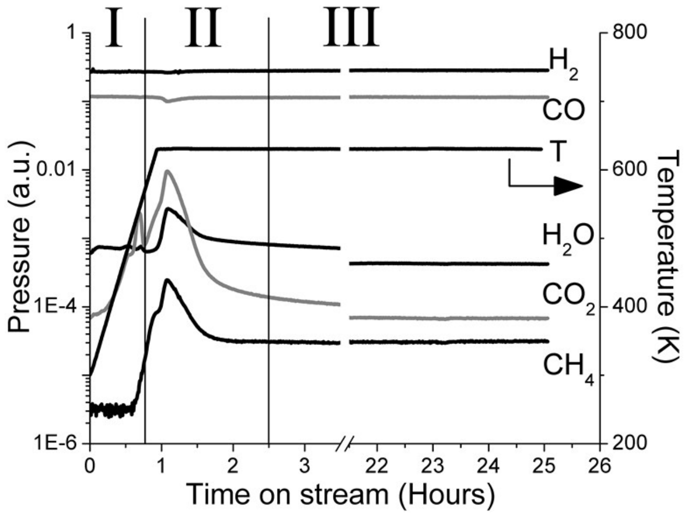

Figure S2 – The reaction profile for micro-reactor CO hydrogenation over the iron oxide catalyst at 623 K for 24 h. The mass traces are labelled on the right hand side in descending order. The Roman numerals indicate the different stages present within the reaction coordinate.

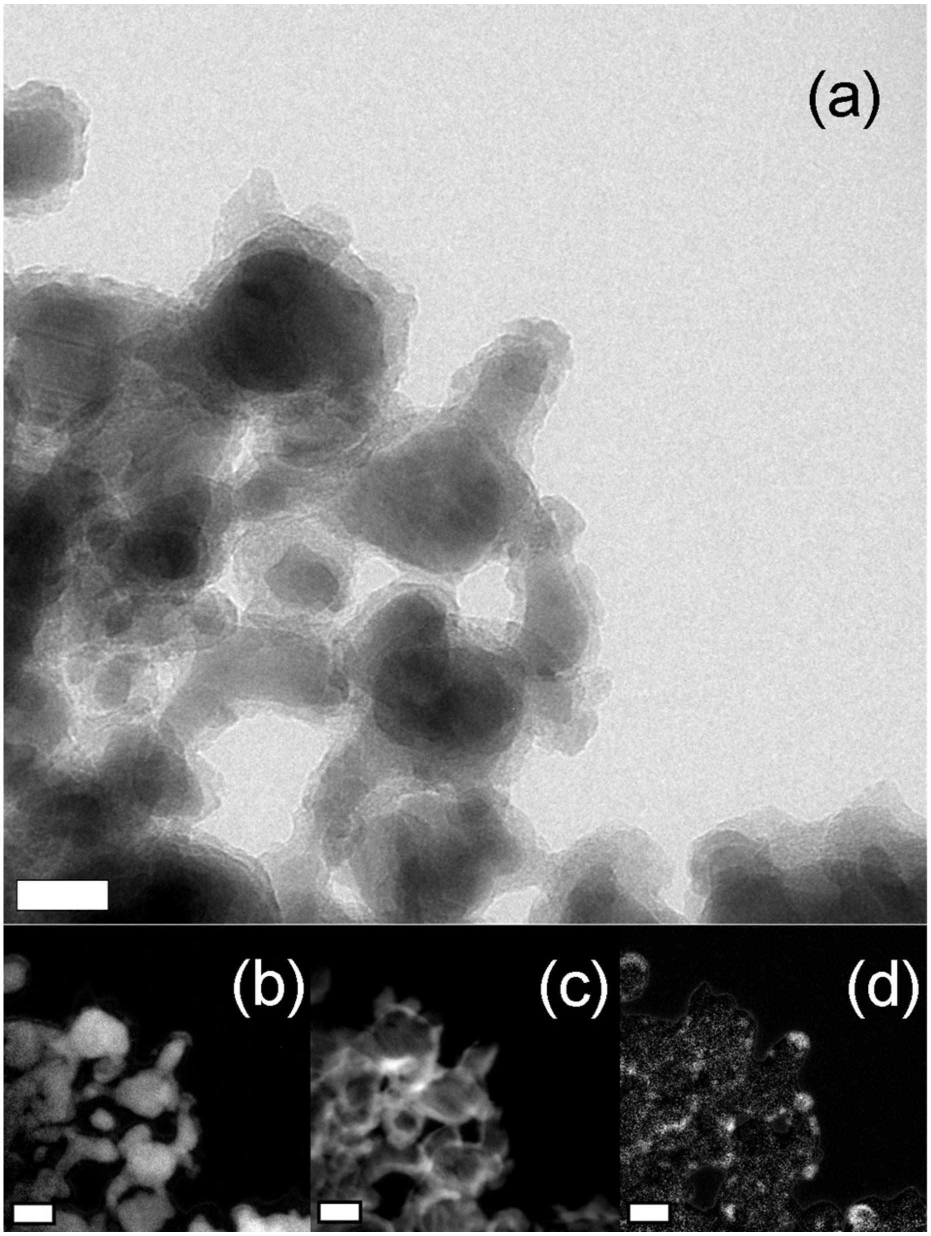

Figure S3 – (a) Ex situ zero energy loss TEM micrograph of the micro-reacted iron oxide catalyst after CO hydrogenation at 623 K for 24 hours with accompanying energy filtered TEM micrographs presenting the (b) iron map, (c) carbon map and (d) oxygen map. The white scale bar in the zero energy loss micrograph indicates a length of 20 nm, whilst it represents a length of 26 nm in the energy filtered micrographs.

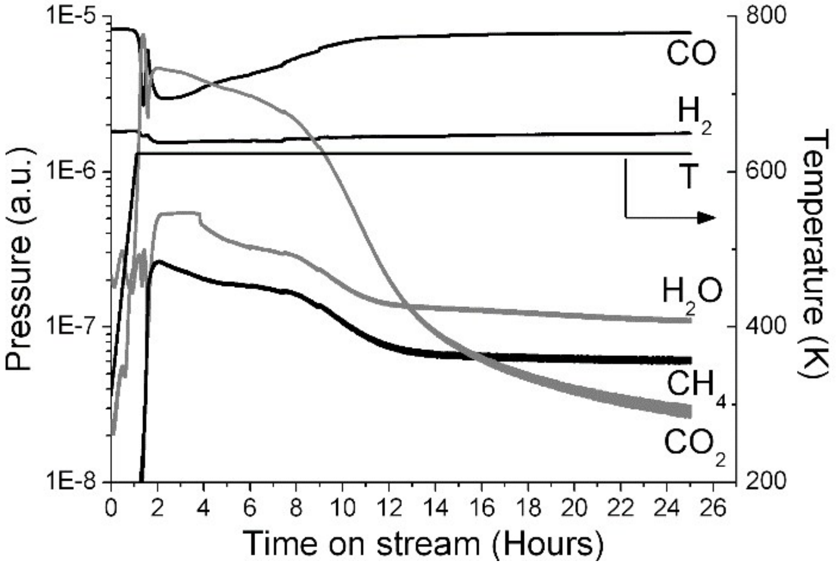

Figure S4 – The reaction profile for scaled-up CO hydrogenation over the iron oxide catalyst at 623 K for 24 h. The mass traces are labelled on the right hand side in descending order.

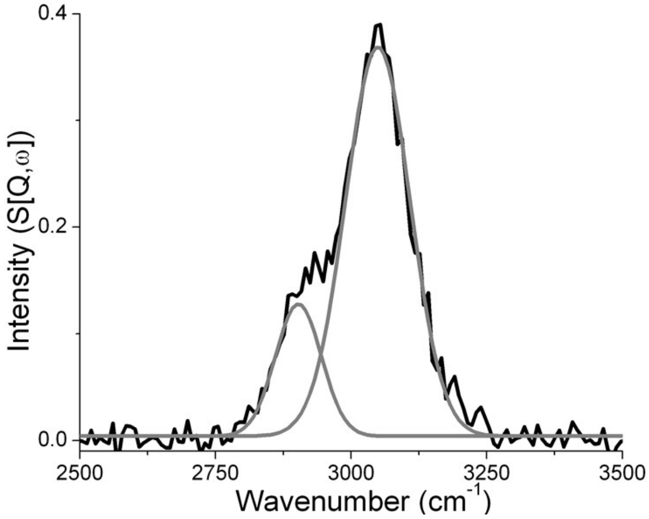

Figure S5 – INS spectra of the iron oxide catalyst after CO hydrogenation at 623 K for 24 hours with Gaussian peak fittings indicating the sp^2 and sp^3 hybridised carbon contributions to the spectra.
